# A method for finding single-nucleotide polymorphisms with allele frequencies in sequences of deep coverage

**DOI:** 10.1186/1471-2105-6-220

**Published:** 2005-09-07

**Authors:** Jianmin Wang, Xiaoqiu Huang

**Affiliations:** 1 Department of Computer Science, Iowa State University, Ames, Iowa 50011, USA

## Abstract

**Background:**

The allele frequencies of single-nucleotide polymorphisms (SNPs) are needed to select an optimal subset of common SNPs for use in association studies. Sequence-based methods for finding SNPs with allele frequencies may need to handle thousands of sequences from the same genome location (sequences of deep coverage).

**Results:**

We describe a computational method for finding common SNPs with allele frequencies in single-pass sequences of deep coverage. The method enhances a widely used program named PolyBayes in several aspects. We present results from our method and PolyBayes on eighteen data sets of human expressed sequence tags (ESTs) with deep coverage. The results indicate that our method used almost all single-pass sequences in computation of the allele frequencies of SNPs.

**Conclusion:**

The new method is able to handle single-pass sequences of deep coverage efficiently. Our work shows that it is possible to analyze sequences of deep coverage by using pairwise alignments of the sequences with the finished genome sequence, instead of multiple sequence alignments.

## Background

Information concerning the allele frequencies of single-nucleotide polymorphisms (SNPs) is needed to select an optimal subset of common SNPs for use in association studies [1]. One approach to finding common SNPs with allele frequencies is to generate DNA sequences from a sufficient number of samples in a population. This approach requires that computational methods have an ability to handle thousands of sequences from the same genome location (sequences of deep coverage). In this paper, we describe a computational method for finding common SNPs with allele frequencies in sequences of deep coverage. We present results from the method on human expressed sequence tags (ESTs) of deep coverage, which are currently a major source of DNA sequences of deep coverage. The method is also expected to be useful for finding common mutations in sequences of deep coverage produced in a cancer genome project [2].

The PolyBayes program is widely used to find SNPs in redundant DNA sequences [3,4]. It first constructs a multiple sequence alignment based on pairwise alignments of each sequence with a high-quality genomic sequence called an anchor. Then it identifies and removes paralogous sequences that have a high number of observed differences with the anchor sequence. Next it computes an SNP probability score for each column of the multiple sequence alignment based on a rigorous Bayesian formula. The formula uses the prior probabilities of all the nucleotide permutations for the column, which are estimated from the quality scores of the bases on the column.

We enhance the PolyBayes program in several aspects to handle single-pass sequences (query sequences) of deep coverage. First, all the paralogous regions of the finished human genome sequence are included as anchor sequences. Each query sequence is assigned to the corresponding anchor sequence that is different from each of the remaining anchor sequences at some positions but is identical to the query sequence at most of the positions. This approach separates paralogous sequences by making use of the positions where paralogous sequences differ but sequences from the same genome location agree.

Second, pairwise alignments of corresponding query and anchor sequences are used to construct profiles, one per anchor sequence. At each position of an anchor sequence, its profile contains the numbers and types of high-quality query bases that are aligned to the position of the anchor sequence. Candidate SNPs are produced based on the profiles, instead of multiple sequence alignments for the following reason. As the number of single-pass sequences in a multiple sequence alignment increases, the number of gap columns in the alignment increases but the number of identity columns in the alignment does not increase. Thus, it is difficult to construct an accurate multiple sequence alignment for single-pass sequences of deep coverage.

Third, because the pairwise alignment of corresponding query and anchor sequences may contain regions of low similarity due to sequencing errors or contaminants, the highly similar regions of the alignment are found by a dynamic programming algorithm. Only the highly similar regions are used in generation of the profile.

Our computer program named PolyFreq was compared with PolyBayes on eighteen data sets of human EST sequences of deep coverage. Results from PolyFreq and PolyBayes indicate that PolyFreq ran to completion and used almost all input sequences in computation of the allele frequencies of SNPs for every data set.

## Results

The method for finding SNPs with allele frequencies was implemented as a computer program. The source code of the program is freely available for academic use [5, see Additional file 1]. The program takes as input a set of high-quality anchor sequences and a set of query sequences with quality scores. The set of anchor sequences includes all the paralogous regions of the genome for the set of query sequences. The anchor and query sequences are from the same species.

The program reports candidate SNPs in the anchor sequences. For each candidate SNP, the program reports its position in the anchor sequence, its local context in the anchor sequence, and base types with a frequency greater than a cutoff. The frequency of a base type is also given in a rational form with the number of query bases of the type as the enumerator and the total number of query bases as the denominator.

To evaluate PolyFreq, eighteen data sets of human EST sequences of deep coverage were constructed as follows. Eighteen clusters of human EST sequences, each containing at least 1,000 EST sequences with trace data, were randomly selected from the April, 2005 release of the UniGene database [6]. The eighteen UniGene clusters also contain EST sequences without trace data. For each of the eighteen UniGene clusters, an EST data set was obtained by selecting all EST sequences with trace data from the cluster. The set of quality score sequences for each of the eighteen data sets was produced from the trace data with Phred [7]. The quality score *q *of a base is obtained by the formula *q *= -10 log *p*, where *p *is the estimated error probability of the base [8]. For example, a quality score of 20 corresponds to an error probability of 0.01. The EST sequences in each of the eighteen sets were produced from 71 to 118 cDNA libraries derived from diverse human tissues and cell lines [9]. Each of the eighteen data sets of full-length EST sequences without any masking was used as a query set.

For each query set, its set of anchor sequences was obtained by comparing the query sequences with the finished genome sequence at the BLAT web server [10]. By using stringent settings for BLAT, a set of two human anchor sequences was produced for each of three query data sets, and a set of one human anchor sequence was produced for each of the remaining query data sets. Each set of anchor sequences was screened for repeats with RepeatMasker [11].

The PolyFreq program was run on each pair of query and anchor sets. The PolyFreq program ran successfully to completion for each of the eighteen data sets. The following values were used for the parameters of the program: 50, minimum depth of coverage for each candidate SNP; 0.1%, minimum minor allele frequency; 5 bp, minimum perfect block length; 20, minimum base quality score in the perfect block; 90%, minimum percent identity for query-anchor alignments; 97%, minimum percent identity for the highly similar regions of query-anchor alignments.

Although PolyBayes was not designed to deal with data sets of deep coverage, we tested PolyBayes on the eighteen data sets of deep coverage to see how PolyBayes would behave on the data sets. Because PolyBayes takes only one anchor sequence, the corresponding anchor sequence was selected and given to PolyBayes for each data set. On eight of the eighteen data sets, PolyBayes ran successfully to completion. On the remaining data sets, PolyBayes terminated abnormally without producing any output file after running for a few hours. The default values for all the parameters but the SNP probability output cutoff of PolyBayes were used; PolyBayes terminated abnormally more frequently under other parameter values. A value of 0.75 for the SNP probability output cutoff was used to produce a lower number of false positives than the default value of 0.5.

The abnormal termination of PolyBayes might be related to the deep coverage of the data set and full-length EST sequences with low-quality ends or contaminants. Thus, for each set of full-length EST sequences, a set of trimmed EST sequences was produced by removing the ends of every sequence that are not highly similar to the corresponding anchor sequence. For each data set, the number of trimmed sequences was close to the number of full-length sequences. The PolyBayes program was also run on each set of trimmed sequences. It ran to completion for thirteen out of the eighteen data sets.

All the tests were performed on a Dell workstation with two 3.0-Ghz processors and 4 Gb of main memory. PolyFreq took less than one hour on every data set, whereas PolyBayes was two to ten times slower than PolyFreq on every data set. The memory requirements of PolyFreq and PolyBayes on the data sets were similar and were about 30 to 40 times the input size.

The PolyFreq and PolyBayes programs were compared on every data set for which PolyBayes ran successfully to completion on either the set of full-length sequences or the set of trimmed sequences. For each data set, results produced by PolyFreq on the set of full-length sequences were included in the comparison, whereas results produced by PolyBayes on both sets of full-length and trimmed sequences were included. The SNPs from the dbSNP database [12] that were mapped by the following method to the anchor sequences were used as true SNPs for the comparison. Each SNP from dbSNP is specified by a local sequence context. For each data set of EST sequences with a RefSeq sequence [13], each SNP from dbSNP that occurs in the RefSeq sequence was determined by finding the exact occurrence of its sequence context in the RefSeq sequence. Each SNP from dbSNP in the RefSeq sequence was mapped to a corresponding anchor sequence by using a spliced alignment of the RefSeq and anchor sequences. Because four data sets had no RefSeq sequence, no SNPs from dbSNP were mapped to the anchor sequences for the data sets.

For each program on every data set with a RefSeq sequence, the number of true positives, the number of false positives, and the number of false negatives were computed. The number of true negatives was not collected because of its large value. Also reported were the number of sequences in the data set and the number of sequences that were used by the program to compute candidate SNPs. The comparison results are shown in Table 1.

**Table 1:** Results by PolyFreq and PolyBayes on eighteen data sets of EST sequences

Data set	Size	PolyBayes (trimmed)				PolyFreq (full length)				PolyBayes (full length)			
		
		TP	FP	FN	NSU	TP	FP	FN	NSU	TP	FP	FN	NSU
Hs.119589	4403	12	170	50	1491	5	24	57	4391	12	152	50	1531
Hs.129673	1665	7	48	11	1457	5	11	13	1662	T/A			
Hs.148340	1603	3	36	9	1563	4	9	8	1583	3	67	9	1560
Hs.170622	1514	1	37	12	429	3	18	10	1507	2	73	11	365
Hs.178551	1685	5	62	12	1632	6	14	11	1676	T/A			
Hs.180909	1017	3	50	6	983	3	20	6	1012	3	164	6	996
Hs.187199	2041	T/A					N/A		1997	T/A			
Hs.198281	3156	9	110	22	3077	15	54	16	3149	T/A			
Hs.350927	1017	5	42	11	976	9	21	7	1015	6	139	10	995
Hs.356331	1441	2	55	9	318	1	14	10	1436	2	85	9	239
Hs.356572	2822	0	46	2	2534	0	17	2	2821	T/A			
Hs.439552	7163	T/A					N/A		6873	T/A			
Hs.444467	4033		N/A		805		N/A		4028		N/A		679
Hs.446628	1490	4	32	12	1338	5	12	11	1486	T/A			
Hs.520640	4120	T/A				9	52	27	4099	T/A			
Hs.522463	8294	T/A					N/A		8280	T/A			
Hs.524390	4462	T/A				9	48	24	4454	T/A			
Hs.544577	7537	14	60	43	1716	10	17	47	7517	18	175	39	1556

The mark N/A means that no SNP from dbSNP was mapped to the anchor sequence because of lack of a RefSeq sequence. The mark T/A means that PolyBayes terminated abnormally without producing any output file. A candidate SNP from the program is considered as true positive (TP) if it is in dbSNP or false positive (FP) otherwise. A SNP from dbSNP that occurs in the data set is considered as false negative (FN) if it is not reported as a candidate SNP from the program. The number of sequences used (NSU) by the program in generation of candidate SNPs is reported.

The results in Table 1 indicate that PolyFreq could handle the data sets of full-length reads with problem regions and with very deep coverage. PolyFreq used 1,997 to 8,280 sequences on the five data sets for which PolyBayes terminated abnormally. On the data sets for which PolyBayes ran to completion, PolyFreq was similar to PolyBayes in the number of true positives and the number of false negatives, and is better than PolyBayes in the number of false positives. PolyBayes used significantly fewer sequences than PolyFreq on some of the data sets. Note that the ability to use as many sequences as possible is necessary for accurate computation of the allele frequencies of SNPs.

## Discussion

We originally developed a method for assembling sequences of deep coverage. The method constructs multiple sequence alignments of large width for contigs. The method has to deal with a large number of gap columns in the multiple sequence alignment. We later agreed with one of the reviewers that it is not necessary to construct multiple sequence alignments for analysis of sequences of deep coverage. The reviewer also suggested that we focus on SNP analysis in sequences of deep coverage. Those suggestions motivated us to develop the method reported in this paper.

The PolyFreq program keeps PolyBayes' feature of performing comparisons between query and anchor sequences, instead of performing comparisons among query sequences. In addition, PolyFreq constructs profiles by using the highly similar regions of pairwise alignments of corresponding query and anchor sequences, instead of multiple alignments of query and anchor sequences. Thus, the efficiency and accuracy of PolyFreq are not significantly affected by query sequences of deep coverage. On the contrary, PolyFreq can compute the allele frequencies of SNPs more accurately in query sequences of deep coverage.

As sequencing costs are significantly reduced in the future, single-pass sequences from hundreds to thousands of individuals will be produced. Those sequences will be of deep coverage. Our current work suggests that it is possible to analyze sequences of deep coverage by using pairwise alignments of the sequences with the finished genome sequence, instead of multiple sequence alignments.

## Methods

We first present the major steps of our method for finding common SNPs with allele frequencies in a set of query sequences and a set of anchor sequences. Then we describe each step in detail. The method consists of the following major steps:

1. Compute an alignment of anchor sequences for each pair of anchor sequences.

2. Compute an alignment of query and anchor sequences for each pair of similar query and anchor sequences.

3. For each query sequence, find the corresponding anchor sequence that is different from each of the remaining anchor sequences at some positions but is identical to the query sequence at most of the positions.

4. Find the highly similar regions of their alignment for each pair of corresponding query and anchor sequences.

5. For each anchor sequence, use the highly similar regions of every alignment involving the anchor sequence to construct a profile for the anchor sequence. At each position of the anchor sequence, its profile contains the numbers and types of high-quality query bases that are aligned to the position of the anchor sequence.

6. Report each candidate SNP with major and minor allele frequencies if its minor allele frequency is greater than a cutoff.

In step 1, for each pair of anchor sequences, an alignment of the sequences in given orientation as well as an alignment of the sequences in opposite orientation is constructed with GAP3, a global alignment program specially designed for genomic sequences with long different regions between similar regions [14]. One of the two alignments with a larger score is saved for the pair of sequences. The alignments saved in this step are to be used in step 3 for finding the corresponding anchor sequence for each query sequence.

In step 2, pairs of similar query and anchor sequences are found with DDS2, which produces a high-scoring chain of segment pairs (ungapped alignment fragments) between the two sequences in the pair [15]. For each pair of similar query and anchor sequences, an alignment of the sequences in the pair is constructed with GAP22, an improved version of the GAP2 program [16] for quickly computing an alignment in a small area of the dynamic programming matrix, which is determined based on the chain of segment pairs. If the percent identity of the alignment is greater than a cutoff, then the alignment is saved for the pair of sequences.

In step 3, for each query sequence that is highly similar to two or more anchor sequences, the corresponding anchor sequence for the query sequence is selected among the anchor sequences through pairwise comparisons. Initially, one anchor sequence is taken as the current leader. Then the rest are compared with the current leader one at a time. Consider the comparison between the current leader and the current challenger. The winner between the two anchor sequences is produced by using the alignment of the two anchor sequences and their alignments with the query sequence. A common match occurs at a position of the query sequence, a position of the current leader, and a position of the current challenger if the three positions are pairwise aligned on each of the three alignments and contain the same base. The winner between the two anchor sequences is the one with a larger number of uncommon matches in its alignment with the query sequence. The winner becomes the current leader. After all the pairwise comparisons, the final leader is the corresponding anchor sequence for the query sequence.

In step 4, for each pair of corresponding query and anchor sequences, the highly similar regions of the alignment of the two sequences are identified in linear time with LCP, a program for finding regions of a sequence that meet a content requirement [17]. Each of the highly similar regions found by LCP has a percent identity greater than or equal to a cutoff *p *and is strictly optimal. The score of a region of the alignment is the sum of scores of every base match and every base difference in the region, where the score of every base match is 1 - *p *and the score of every base difference is - *p*. A region is optimal if its score is not less than the score of any other region that overlaps with it. An optimal region is strictly optimal if it is not completely contained in any optimal region other than itself.

**Figure 1:**
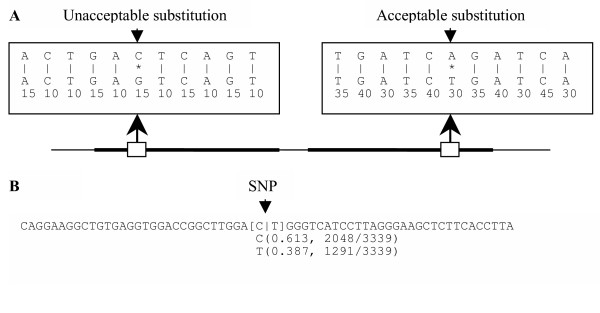
Acceptable and unacceptable substitutions in a pairwise alignment and a candidate SNP from a real data set. (**A**) The line shows an alignment of query and anchor sequences with thick parts indicating highly similar regions. The large rectangular box gives a detailed view of the small box. On the left is an unacceptable substitution that is flanked by a block of low-quality bases, and on the right is an acceptable substitution that is flanked by a perfect block on each side. The quality value of each query base in the large box is shown next to the base. (**B**) Shown is a candidate SNP with allele frequencies from PolyFreq on a real data set (Hs. 119589) in Table 1.

In step 5, only substitutions in the highly similar regions of every alignment of corresponding query and anchor sequences are used to construct a profile for the anchor sequence because the remaining regions of the alignment have a high rate of difference, which is likely due to sequencing errors or contaminants in the query sequence. Additional requirements are introduced below because a long highly similar region may still contain a packet of sequencing errors in the middle. A sufficiently long section in a highly similar region of an alignment is a perfect block if the section consists only of exact base matches and the quality score of each query base in the section is greater than or equal to a cutoff [18]. A substitution in a highly similar region of an alignment is acceptable if it is immediately flanked on each side by a perfect block. Acceptable and unacceptable substitutions are illustrated in Figure 1A.

For each anchor sequence, its profile contains four counts at each position: one count for each query base type. For example, the base A count at the anchor position is the number of acceptable substitutions at the anchor position and at a query sequence position with base A, in a highly similar region of an alignment of the anchor sequence with the query sequence. A count for a query base type at the anchor position is 0 if there is no acceptable substitution at the anchor position and at any query sequence position with the query base type. The frequency of each of the four counts is the count divided by the sum of the four counts if the sum is positive.

In step 6, each profile is scanned for candidate SNPs. A candidate SNP occurs at an anchor sequence position if the sum of the four counts for the position is greater than or equal to a cutoff and at least two of the four counts have a frequency greater than a cutoff. All candidate SNPs with allele frequencies are reported along with a local anchor sequence region for each candidate SNP. A candidate SNP with allele frequencies from one of the examples in Table 1 is shown in Figure 1B.

## Author's contributions

XH designed the strategy for solving the problem and provided guidance to JW. JW worked out the details of the strategy, developed the program, and produced results on data sets with the program. XH wrote the paper and JW formatted it in Word. All authors read and approved the final manuscript.

## Supplementary Material

Additional File 1 A file named PolyFreq.tar is included. The file contains the source code of all programs in the package. The file is unpacked by using the Unix command "tar xvf PolyFreq.tar" on a Unix or Linux computer. Click here for file
